# Access-to-care: evidence from home-based postnatal coordinated care after hospital discharge

**DOI:** 10.1186/s12913-021-07151-3

**Published:** 2021-10-22

**Authors:** Saad Zbiri, Patrick Rozenberg, Carine Milcent

**Affiliations:** 1 EA 7285, Versailles Saint-Quentin University, Montigny-le-Bretonneux, France; 2 International School of Public Health, Mohammed VI University of Health Sciences, Casablanca, Morocco; 3 Department of Obstetrics and Gynecology, Poissy Saint-Germain Hospital, Poissy, France; 4 French National Center for Scientific Research (CNRS), Paris, France; 5 Paris School of Economics (PSE), Paris, France

**Keywords:** Postnatal care, Home-based coordinated care, Patient information, Health professional accessibility, Inequity

## Abstract

**Background:**

Home-based postnatal care after hospital discharge has become an integral part of postnatal care. This study aimed to determine the factors relating either to individuals or the healthcare system that affect enrollment and full participation (adherence) in the French home-based postnatal coordinated care program (PRADO).

**Methods:**

All admitted women for delivery in a French district over one year and eligible for this home-based midwifery support after hospital discharge were included (*N* = 4189). Both a simple probit model and a probit Heckman selection model were used. The control variables were the characteristics of the women, the municipalities, and the hospitals.

**Results:**

Approximately 68% of the eligible women chose to enroll in the PRADO program, of who nearly 60% fully participated in this program. Enrollment in the program was influenced mostly by the family context, such as the woman’s age at the time of her pregnancy and the number of children in the household, the woman’s level of prenatal education and information about postnatal care, as well as some hospital variables such as the characteristics and organization of the maternity units. Full participation in the program was influenced by the accessibility to health professionals, particularly midwives. Furthermore, the women’s level of prenatal education and information about postnatal care, as well as their accessibility to health professionals, correlated with the socioeconomic environment.

**Conclusion:**

While individual factors impacted enrollment in the PRADO program, only healthcare system-related factors influenced full participation in the program. A public health policy promoting home-based postnatal care could increase the women’s participation by improving their level of prenatal education and information about postnatal care. In addition, reducing regional inequality is likely to have a positive impact, as the availability of health professionals is a key factor for participation in home-based postnatal coordinated care.

**Supplementary Information:**

The online version contains supplementary material available at 10.1186/s12913-021-07151-3.

## Background

Postnatal care after delivery has come to include two consecutive components, namely inpatient hospital care followed by home-based care. Therefore, early postpartum care no longer solely comprises hospital care, as was previously the case. In France, a home-based postnatal coordinated care after hospital discharge has been introduced [1].

There has been a gradual decrease in the length of hospital stay following delivery in most high-income countries over the past 40–50 years as a result of medical, economic, social, cultural, and political changes [2, 3]. The main factors underlying this trend of reduced reliance on postnatal care in the hospital setting include increased efforts to reduce the risk of nosocomial infections, stress, and sleeping disorders for women and their newborns [4], to improve satisfaction during this period by supporting parental requests to return home soon after the childbirth [5, 6], and to limit the cost to the healthcare system [7].

A consensus has not been reached to date regarding the appropriate length of postpartum stay in the hospital. The World Health Organization (WHO) recommends that all women should remain in the hospital at least 24 h after childbirth. This recommendation is mainly intended for low-income countries, as it allows identification of any serious complications that require emergency care [8]. However, postpartum care is an important preventive support that should not be limited to the immediate postpartum period. It allows monitoring and treatment of complications in the mother and/or the newborn, the provision of support to facilitate the woman’s transition to going home, counseling regarding infant feeding, parent education processes for newborn care, and health promotion indications [9]. Although it varies considerably, the average length of the hospital stay for low-risk mothers and newborns recommended in several high-income countries ranges from 2 to 3 days [1, 2, 10].

The current trend of healthcare services to encourage early hospital discharge of women following delivery has led to a number of concerns. These are centered on the notion that hospitals may not be providing sufficient appropriate services or infrastructure support to women as a result of this trend of early discharge from the hospital after childbirth. Reduction in the length of stay underscores the need for home-based postnatal coordinated care to support women who are discharged from the hospital before they have received adequate education regarding being a new parent. In some countries, it is recommended that postnatal management should incorporate well-established home-based postnatal coordinated care after discharge from the hospital [1, 10–12]. However, while hospital care is provided to all admitted patients, some women may end up not continuing with the postpartum coordinated care once they have been discharged.

Our study objective was to identify the determinants of the use, both in terms of enrollment and full participation (adherence), of home-based postnatal coordinated care after being discharged from the hospital. We focused not only on patient-level variables including household characteristics and care characteristics but also on factors related to the hospitals and to the healthcare system. Our study tested the hypothesis that specific patient, municipality, and/or hospital characteristics can impact the initial enrollment and/or full participation in home-based postnatal coordinated care. This study, therefore, adds considerably to the otherwise rather scarce literature regarding access to postnatal care. Two sources of key factors that affect the use of home-based postnatal care after hospital discharge were identified. These were, on the one hand, the individual factors of enrollment and, on the other hand, the elements of the healthcare system that limited full participation even when the woman enrolled.

## Methods

### Data source

This was a French population-based retrospective study of women eligible for coordinated postnatal care after discharge from the hospital. Since 2010, the single-payer public health insurance system has provided home-based coordinated care to support women once they have been discharged from the hospital after having given birth. First, a public health insurance advisor at the maternity ward assists the woman with getting in touch with a midwife. If the woman is interested in participating in the home-based postpartum coordinated care program (PRADO), 2 visits are scheduled between day 0 (day of delivery) and day 7, and the first visit has to be on the first day after the woman’s return home. The hospital discharge under the PRADO program depends on the approval of the maternity medical team (doctor or midwife).

The data were from 3 linked administrative datasets for the year 2013. These 2013 datasets allowed us to have access to detailed and verified information, which was not possible with more recent data. The main dataset was extracted from the public health insurance dataset of the Yvelines district, which contains all of the insured people of the district (100% of the population). The data comprised the information regarding the women’s individual demographic, socioeconomic, and location data, as well as the characteristics of the prenatal and the postnatal care of the women, and information regarding the hospital of admission. The second dataset, compiled by the French National Institute of Statistics, was extracted from population census data and it provided information regarding the socioeconomic situation of all French municipalities. The third dataset, compiled by the Ministry of Health, provided information regarding the accessibility to health professionals for all French municipalities. The 3 datasets were merged using the denomination and the code of the municipally of residence of the women.

### Study population

The study population consisted of all women affiliated with the public health insurance agency of the Yvelines district and who were eligible for participation in the PRADO program (*N* = 4189). To be eligible, women had to be at low clinical risk and admitted to a hospital for delivery: women aged 18 years and older, without any co-morbidity or complication, giving birth by vaginal delivery at full term to a singleton infant not requiring maintenance in a medical environment nor a particular feeding mode.

### Study variables

Our variables included individual characteristics of the women, municipality characteristics, and the hospital characteristics and organization.

The patient-level variables considered were age (5 age brackets: 18 to 23 years, 24 to 29 years, 30 to 35 years, 36 to 41 years, and 42 years or older), the number of children in the household (4 categories: 1, 2, 3, and 4 children or more), the type of healthcare coverage (2 categories: policyholder and person eligible for benefits, referred to here as the beneficiary), the number of antenatal visits (3 categories: 0 to 5, 6 or 7, and 8 visits or more), prenatal follow-up by a gynecologist (2 categories: yes and no, “yes” if at least 1 antenatal visit), prenatal follow-up by a general practitioner (2 categories: yes and no, “yes” if at least 1 antenatal visit), prenatal follow-up by a midwife (2 categories: yes and no, “yes” if at least 1 antenatal visit), prenatal hospital follow-up (2 categories: yes and no, “yes” if at least 1 antenatal visit), prenatal community follow-up (2 categories: yes and no, “yes” if at least 1 antenatal visit), the number of obstetric ultrasounds (3 categories: 0 or 1, 2 or 3, and 4 ultrasounds or more), prenatal education (2 categories: yes and no), prenatal information regarding postnatal care (2 categories: yes and no), and postnatal hospital readmission of the woman or the newborn until day 7 (2 categories: yes and no). The municipality-level variables considered were the location of the municipality (2 categories: urban and rural), household deprivation as assessed by the median annual income (4 categories at regular intervals based on the variable values and not the variable distribution in the population: least deprived (from 37,300 to 46,100 euros), less deprived (from 28,500 to 37,299 euros), more deprived (from 19,700 to 28,499 euros), and most deprived (from 10,900 to 19,699 euros), accessibility to a gynecologist as assessed by the index of spatial accessibility (ISA) (4 categories at regular intervals based on the variable values: lowest (1 to 4.7), low (4.8 to 8.5), high (8.6 to 12.3), and highest (12.4 to 16)), accessibility to a general practitioner as assessed by the ISA (4 categories at regular intervals based on the variable values: lowest (7 to 31.9), low (32 to 56.9), high (57 to 81.9), and highest (82 to 106)), and accessibility to a midwife as assessed by the ISA (4 categories at regular intervals based on the variable values: lowest (1 to 2.4), low (2.5 to 3.9), high (4 to 5.4), and highest (5.5 to 7)). The hospital-level variables considered were funding of the hospital (2 categories: public and private), hospital university status (2 categories: teaching and non-teaching), level of care of the maternity ward (3 categories: no neonatology unit, neonatology unit, and neonatal intensive care unit), staffing levels for obstetricians as measured by the number of obstetricians in full-time equivalents (FTEs) per 100 deliveries, staffing levels for midwives as measured by the number of midwives in FTEs per 100 deliveries, the day of delivery (2 categories: working day and non-working day), and the day of hospital discharge (2 categories: working day and non-working day).

The index of spatial accessibility (ISA) used here was developed by the French Ministry of Health. This index provides a measure of the spatial adequacy between supply and demand for primary care at a local level. It is captured, on the supply side, by the density of health professionals while taking into account their practice rates in the health area and, on the demand side, by the geographical distance between patients and the professionals in the zone defined above while considering differences in patient age groups and in the observed rates of attendance by professionals [13]. This index is based on the “two-step floating catchment area” method and is interpreted as a density [14].

### Statistical analysis

We studied the main factors that affect the use of the PRADO program. As a first model, we assessed the probability of choosing to participate in the PRADO program. Using a probit regression, we estimated the probability of the initial enrollment in the PRADO program for all of the eligible women as a function of the household and prenatal care characteristics, the municipality characteristics, as well as the hospital characteristics and organization. Enrollment was defined as a dummy variable equal to 1 with enrollment in the PRADO program and equal to 0 when the woman did not wish to participate in the PRADO program.

We then estimated the probability of full participation in the PRADO program. Full participation in the coordinated program was defined as agreeing to partake in the 2 home-based postnatal visits, with the first visit in the first 24 h after being discharged. Full participation was defined as a dummy variable equal to 1 with full participation in the PRADO program and equal to 0 when the woman did not partake in the entire PRADO program. As the independent variables, we considered a vector of explanatory variables with household characteristics, prenatal care variables, postnatal hospital readmission, and the municipality characteristics including indicators of the accessibility to health professionals. First, we studied the population of women enrolled in this postpartum care program. In the sample, 68% of the patients eligible for this program decided to participate. We thus estimated a probit regression for the probability of full participation in the PRADO program (second model). The results obtained here were conditional on the decision to participate. In case the selection process to enroll or not to enroll was non-exogenous, we used a Heckman probit model to assess the probability of full participation in the PRADO program (third model). We assumed that the hospital characteristics affect the decision to participate but that they do not affect full participation once the woman has decided to participate in the program [15].

Finally, when relevant, we measured the correlation between any determinant factor(s) that significantly impacted the initial enrollment and/or full participation in the PRADO program and the socioeconomic environment as measured by the median annual income of the municipality.

All probit models were performed using the cluster-robust variance that accounted for the dependence between the observations at the municipality level. The results are reported as coefficients with their 95% confidence intervals (CIs). The significance levels are two-sided with a probability threshold of *p* < 0.05. The analyses were performed using Stata software (Stata Corporation, College Station, TX, USA) [16].

## Results

A total of 4189 eligible women were included in the analysis. Of these, 2859 women chose to participate in the PRADO program after their discharge from the hospital, thus amounting to 68.3% of the total (95% CI [66.8;69.6]). Of the women who opted to participate in the PRADO program, 2496 participated in the full PRADO program, thus amounting to 87.3% (95% CI [86.0;88.5]) of the women who chose to participate and 59.6% (95% CI [58.1;61.1]) of the total number of eligible women. Table 1 presents the descriptive statistics.

**Table 1:** Descriptive characteristics of the study population

	Women opting to participate in the PRADO program	All of the women eligible for the PRADO program
	(*n* = 2859)	(*n* = 4189)
**Household characteristics**		
Woman’s age at pregnancy (years), n (%)		
18–23	176 (6.16)	312 (7.45)
24–29	978 (34.21)	1413 (33.73)
30–35	1196 (41.83)	1711 (40.85)
36–41	473 (16.54)	685 (16.35)
≥ 42	36 (1.26)	68 (1.62)
Number of children, n (%)		
1	1720 (60.16)	2348 (56.05)
2	713 (24.94)	1056 (25.21)
3	286 (10.00)	518 (12.37)
≥ 4	140 (4.90)	267 (6.37)
Woman’s healthcare coverage, n (%)		
Policyholder	2625 (91.82)	3751 (89.54)
Beneficiary	234 (8.18)	438 (10.46)
**Prenatal care**		
Antenatal visits, n (%)		
0–5	769 (26.90)	1205 (28.77)
6–7	666 (23.29)	974 (23.25)
≥ 8	1424 (49.81)	2010 (47.98)
Follow-up by a gynecologist^a^, n (%)		
No	765 (26.76)	1156 (27.60)
Yes	2094 (73.2)	3033 (72.40)
Follow-up by a general practitioner^a^, n (%)		
No	835 (29.21)	1256 (29.98)
Yes	2024 (70.79)	2933 (70.02)
Follow-up by a midwife^a^, n (%)		
No	1257 (43.97)	1907 (45.52)
Yes	1602 (56.03)	2282 (54.48)
Hospital follow-up^a^, n (%)		
No	898 (31.41)	1309 (31.25)
Yes	1961 (68.59)	2880 (68.75)
Community follow-up^a^, n (%)		
No	388 (13.57)	605 (14.44)
Yes	2471 (86.43)	3584 (85.56)
Obstetric ultrasound, n (%)		
0–1	761 (26.62)	1213 (28.96)
2–3	1346 (47.08)	1913 (45.67)
≥ 4	752 (26.30)	1063 (25.38)
Prenatal education, n (%)		
No	1583 (55.37)	2631 (62.81)
Yes	1276 (44.63)	1558 (37.19)
Prenatal information regarding postpartum, n (%)		
No	2416 (84.51)	3709 (88.54)
Yes	443 (15.49)	480 (11.46)
**Postnatal care**		
Hospital readmission, n (%)		
No	2811 (98.32)	4123 (98.42)
Yes	48 (1.68)	66 (1.58)
**Municipality characteristics**		
Location, n (%)		
Urban	2647 (92.58)	3890 (92.86)
Rural	212 (7.42)	299 (7.14)
Household deprivation^b^, n (%)		
Least deprived	70 (2.45)	96 (2.29)
Less deprived	745 (26.06)	971 (23.18)
More deprived	1371 (47.95)	1966 (46.93)
Most deprived	673 (23.54)	1156 (27.60)
Accessibility to a gynecologist^c^, n (%)		
Lowest	560 (19.59)	819 (19.55)
Low	1771 (61.94)	2661 (63.52)
High	439 (15.36)	602 (14.37)
Highest	89 (3.11)	107 (2.55)
Accessibility to a general practitioner^c^, n (%)		
Lowest	73 (2.55)	104 (2.48)
Low	1091 (38.16)	1526 (36.43)
High	1166 (40.78)	1677 (40.03)
Highest	529 (18.50)	882 (21.06)
Accessibility to a midwife^c^, n (%)		
Lowest	78 (2.73)	118 (2.82)
Low	1518 (53.10)	2275 (54.31)
High	1101 (38.51)	1588 (37.91)
Highest	162 (5.67)	208 (4.97)
**Hospital characteristics**		
Funding, n (%)		
Public	2398 (83.88)	3531 (84.29)
Private	461 (16.12)	658 (15.71)
University status, n (%)		
Non-teaching	1423 (49.77)	2303 (54.98)
Teaching	1436 (50.23)	1886 (45.02)
Level of care, n (%)		
No neonatology unit	376 (13.15)	611 (14.59)
Neonatology unit	816 (28.54)	1173 (28.00)
Neonatal intensive care unit	1667 (58.31)	2405 (57.41)
Obstetricians^d^, mean ± SD	0.47 ± 0.12	0.48 ± 0.12
Midwives^d^, mean ± SD	1.73 ± 0.31	1.71 ± 0.30
Day of delivery, n (%)		
Working	2776 (97.10)	4082 (97.45)
Non-working	83 (2.90)	107 (2.55)
Day of discharge, n (%)		
Working	2790 (97.59)	4074 (97.25)
Non-working	69 (2.41)	115 (2.75)

^a^ At least one antenatal visit

^b^ Based on the median annual income

^c^ Based on the index of spatial accessibility (ISA)

^d^ FTEs (full-time equivalents) per 100 deliveries

Table 2 shows the determinants of the initial enrollment in the PRADO program for all of the eligible women. We found several groups of observable factors that were significantly associated with enrollment in the PRADO program. The first group of factors was the household characteristics: women with a pregnancy at a very early or late stage of their life, those having 3 children or more, and women who were not health insurance policyholders but were eligible for benefits (referred to here as a beneficiary) had a lower probability of enrollment in the PRADO program. These variables, especially the healthcare coverage variable, may capture socioeconomic aspects such as occupation or employment status. The second group of factors was the prenatal and postnatal information collected: participation in a prenatal education program and participation in a communication program for prenatal information on postpartum care. These programs aimed at providing information are organized and presented by the medical staff during the pregnancy. We found that the women who attended a prenatal education program and those who had received information regarding postnatal care were more likely to enroll in the PRADO program. The third group of factors was the hospital unit characteristics: women admitted to private hospitals, to non-teaching hospitals, to hospitals with a high level of care (based on the presence of a specific neonatology care unit), and to hospitals with a high level of staffing of both obstetricians and midwives (as measured by the FTE appointments per 100 deliveries over the average) had a relatively low probability of enrollment in the program. Furthermore, women who delivered on non-working days and those who were discharged on working days were more likely to enroll in the PRADO program.

**Table 2:** Factors associated with initial enrollment in the home-based postnatal coordinated care (PRADO) for all of the women eligible for this program. Probit regression model (*n* = 4189)

	Coefficient	[95% CI]
**Household characteristics**		
Woman’s age at pregnancy (years)		
18–23	− 0.31^***^	[− 0.44;-0.18]
24–29	− 0.04	[− 0.13;0.05]
30–35	reference	
36–41	0.07	[− 0.04;0.18]
≥ 42	− 0.42^**^	[− 0.69;-0.14]
Number of children		
1	reference	
2	−0.09	[− 0.20;0.02]
3	− 0.28^***^	[− 0.40;-0.17]
≥ 4	− 0.31^***^	[− 0.47;− 0.15]
Woman’s healthcare coverage		
Policyholder	reference	
Beneficiary	-0.15^*^	[−0.28;-0.01]
**Prenatal care**		
Antenatal visits		
0–5	− 0.05	[−0.19;0.09]
6–7	reference	
≥ 8	−0.02	[− 0.13;0.09]
Follow-up by a gynecologist^a^		
No	reference	
Yes	−0.09	[− 0.23;0.06]
Follow-up by a general practitioner^a^		
No	reference	
Yes	0.09	[−0.03;0.21]
Follow-up by a midwife^a^		
No	reference	
Yes	−0.10	[−0.24;0.04]
Hospital follow-up^a^		
No	reference	
Yes	−0.06	[− 0.23;0.11]
Community follow-up^a^		
No	reference	
Yes	−0.07	[− 0.23;0.10]
Obstetric ultrasound		
0–1	− 0.07	[− 0.22;0.08]
2–3	reference	
≥ 4	0.03	[− 0.09;0.14]
Prenatal education		
No	reference	
Yes	0.46^***^	[0.36;0.56]
Prenatal information regarding postpartum		
No	reference	
Yes	0.72^***^	[0.51;0.93]
**Municipality characteristics**		
Location		
Urban	reference	
Rural	0.07	[−0.14;0.28]
Household deprivation^b^		
Least deprived	reference	
Less deprived	0.24	[−0.09;0.56]
More deprived	0.07	[−0.27;0.42]
Most deprived	−0.04	[−0.40;0.32]
Accessibility to a gynecologist^c^		
Lowest	reference	
Low	−0.08	[− 0.24;0.09]
High	0.09	[−0.14;0.33]
Highest	0.53^**^	[0.20;0.86]
Accessibility to a general practitioner^c^		
Lowest	reference	
Low	0.00	[−0.30;0.30]
High	0.06	[−0.26;0.37]
Highest	0.06	[−0.26;0.38]
Accessibility to a midwife^c^		
Lowest	reference	
Low	0.16	[−0.06;0.39]
High	0.15	[−0.09;0.39]
Highest	−0.09	[−0.39;0.21]
**Hospital characteristics**		
Funding		
Public	reference	
Private	−0.39^*^	[−0.73;-0.06]
University status		
Non-teaching	reference	
Teaching	0.41^***^	[0.24;0.59]
Level of care		
No neonatology unit	reference	
Neonatology unit	− 0.43^***^	[− 0.66;-0.19]
Neonatal intensive care unit	−0.48^**^	[− 0.83;-0.12]
Obstetricians^d^	−2.07^**^	[−3.56;-0.57]
Midwives^d^	−0.82^**^	[−1.45;-0.19]
Day of delivery		
Working	reference	
Non-working	0.28^*^	[0.01;0.55]
Day of discharge		
Working	reference	
Non-working	−0.25^*^	[− 0.48;− 0.02]

CI, confidence interval. ^*^: *p* < 0.05; ^**^: *p* < 0.01; ^***^: *p* < 0.001

^a^ At least one antenatal visit

^b^ Based on the median annual income

^c^ Based on the index of spatial accessibility (ISA)

^d^ FTEs (full-time equivalents) per 100 deliveries

These results highlight the conditions that affect the initial enrollment of women in the PRADO program. As a result, the conditions for enrollment in the PRADO program were: 1- the household’s intrinsic characteristics, 2- the extent to which the woman had been informed regarding the delivery and the post-delivery period, 3- the hospital characteristics and organization.

The impact of accessibility to a gynecologist was only observed for women living in areas with a high level of accessibility to a gynecologist. This variable may capture other aspects of the women or the municipality.

Table 3 provides the probit model estimates of the full participation in the PRADO program for the sample of women who opted for the PRADO program. A prenatal education program impacted the probability of full participation. Indeed, the women who participated in a prenatal education program were more likely to fully participate in the postnatal coordinated program. As expected, hospital readmission up to 7 days after childbirth decreased the probability of full participation. In terms of factors related to the healthcare system, we found that the accessibility to midwives impacted the probability of full participation in the PRADO program. Using the index of spatial accessibility (ISA), we observed that increased accessibility to a midwife increased the probability of full participation. Unexpectedly, we found that the number of children did not affect full participation in the program after controlling for healthcare accessibility and the prenatal education program. As robustness checks, presented in Table A1 in the Appendix, we also included the hospital variables, and we found that the characteristics of the hospitals were not associated with the women’s full participation in the PRADO program.

**Table 3:** Factors associated with full participation in the home-based postnatal coordinated care (PRADO) for the women enrolled in this program. Probit regression model (*n* = 2859)

	Coefficient	[95% CI]
**Household characteristics**		
Woman’s age at pregnancy (years)		
18–23	0.01	[− 0.27;0.29]
24–29	0.09	[− 0.04;0.22]
30–35	reference	
36–41	0.05	[− 0.09;0.19]
≥ 42	-0.02	[− 0.47;0.43]
Number of children		
1	reference	
2	−0.07	[− 0.23;0.08]
3	−0.07	[− 0.27;0.13]
≥ 4	− 0.18	[− 0.47;0.10]
Woman’s healthcare coverage		
Policyholder	reference	
Beneficiary	−0.16	[− 0.37;0.06]
**Prenatal care**		
Antenatal visits		
0–5	− 0.01	[− 0.23;0.21]
6–7	reference	
≥ 8	0.12	[− 0.04;0.28]
Follow-up by a gynecologist^a^		
No	reference	
Yes	−0.04	[− 0.17;0.10]
Follow-up by a general practitioner^a^		
No	reference	
Yes	−0.13	[− 0.32;0.06]
Follow-up by a midwife^a^		
No	reference	
Yes	0.00	[−0.16;0.17]
Hospital follow-up^a^		
No	reference	
Yes	0.15	[−0.01;0.31]
Community follow-up^a^		
No	reference	
Yes	0.14	[−0.08;0.36]
Obstetric ultrasound		
0–1	−0.02	[−0.22;0.17]
2–3	reference	
≥ 4	−0.13	[−0.27;0.01]
Prenatal education		
No	reference	
Yes	0.21^**^	[0.06;0.35]
Prenatal information regarding postpartum		
No	reference	
Yes	0.15	[−0.03;0.34]
**Postnatal care**		
Hospital readmission		
No	reference	
Yes	−0.67^***^	[−1.04;-0.30]
**Municipality characteristics**		
Location		
Urban	reference	
Rural	−0.01	[−0.27;0.25]
Household deprivation^b^		
Least deprived	reference	
Less deprived	0.01	[−0.26;0.28]
More deprived	0.09	[−0.24;0.42]
Most deprived	−0.13	[−0.50;0.24]
Accessibility to a gynecologist^c^		
Lowest	reference	
Low	−0.21	[− 0.45;0.03]
High	−0.29	[− 0.66;0.09]
Highest	0.04	[−0.51;0.60]
Accessibility to a general practitioner^c^		
Lowest	reference	
Low	0.26	[−0.15;0.66]
High	0.18	[−0.26;0.61]
Highest	0.38	[−0.12;0.88]
Accessibility to a midwife^c^		
Lowest	reference	
Low	0.58^***^	[0.27;0.90]
High	0.62^***^	[0.25;0.98]
Highest	0.72^**^	[0.21;1.24]

CI, confidence interval. ^*^: *p* < 0.05; ^**^: *p* < 0.01; ^***^: *p* < 0.001

^a^ At least one antenatal visit

^b^ Based on the median annual income

^c^ Based on the index of spatial accessibility (ISA)

^d^ FTEs (full-time equivalents) per 100 deliveries

Thus far, we assumed that the enrollment in the PRADO program was a random process. We then considered the self-selection bias in the decision to participate in the PRADO program. Table 4 shows the results regarding the probability of full participation in the PRADO program for all of the eligible women when taking into account this potential self-selection. We thus regressed a probit Heckman selection model. Taking into account the initial enrollment of women in the PRADO program, we controlled for the same entire set of observable characteristics as used before. The impact of the accessibility to a midwife on full participation in the PRADO program remained statistically significant, that is to say, women with a higher level of accessibility to a midwife were more likely to fully participate in the PRADO program. Moreover, the effect of postnatal readmission to the hospital during the first 7 days was still significant. However, the effect of a prenatal education program was no longer significant after taking into account the self-selection bias. The results of the selection equation are reported in Table A2 in the Appendix. The independent significant variables of the selection equation were quite similar to what was found when the probability of PRADO program enrollment was estimated without accounting for self-selection (Table 2). We did not interpret Rho because its nature is extremely sensitive to model specification.

**Table 4:** Factors associated with full participation in the home-based postnatal coordinated care (PRADO) for all of the women eligible for this program. Probit Heckman regression model allowing self-selection into women enrolled in this program (*n* = 4189)

	Coefficient	[95% CI]
**Household characteristics**		
Woman’s age at pregnancy (years)		
18–23	0.10	[−0.16;0.35]
24–29	0.09	[−0.03;0.21]
30–35	reference	
36–41	0.02	[−0.11;0.16]
≥ 42	0.11	[−0.32;0.54]
Number of children		
1	reference	
2	−0.05	[− 0.20;0.10]
3	0.01	[−0.16;0.19]
≥ 4	−0.05	[−0.33;0.22]
Woman’s healthcare coverage		
Policyholder	reference	
Beneficiary	−0.08	[− 0.28;0.13]
**Prenatal care**		
Antenatal visits		
0–5	−0.01	[−0.20;0.18]
6–7	reference	
≥ 8	0.11	[−0.03;0.25]
Follow-up by a gynecologist^a^		
No	reference	
Yes	0.00	[−0.12;0.12]
Follow-up by a general practitioner^a^		
No	reference	
Yes	−0.14	[−0.30;0.03]
Follow-up by a midwife^a^		
No	reference	
Yes	0.00	[−0.14;0.14]
Hospital follow-up^a^		
No	reference	
Yes	0.15	[0.00;0.29]
Community follow-up^a^		
No	reference	
Yes	0.15	[−0.05;0.35]
Obstetric ultrasound		
0–1	0.00	[−0.18;0.17]
2–3	reference	
≥ 4	−0.13^*^	[−0.26;-0.01]
Prenatal education		
No	reference	
Yes	0.08	[−0.06;0.22]
Prenatal information regarding postpartum		
No	reference	
Yes	0.01	[−0.17;0.19]
**Postnatal care**		
Hospital readmission		
No	reference	
Yes	−0.62^***^	[− 0.98;-0.27]
**Municipality characteristics**		
Location		
Urban	reference	
Rural	−0.01	[−0.25;0.23]
Household deprivation^b^		
Least deprived	reference	
Less deprived	−0.02	[− 0.28;0.23]
More deprived	0.09	[−0.22;0.39]
Most deprived	−0.07	[−0.41;0.26]
Accessibility to a gynecologist^c^		
Lowest	reference	
Low	−0.18	[− 0.40;0.04]
High	−0.28	[− 0.61;0.06]
Highest	−0.05	[− 0.55;0.46]
Accessibility to a general practitioner^c^		
Lowest	reference	
Low	0.24	[−0.14;0.62]
High	0.15	[−0.25;0.55]
Highest	0.36	[−0.10;0.81]
Accessibility to a midwife^c^		
Lowest	reference	
Low	0.48^**^	[0.17;0.80]
High	0.53^**^	[0.18;0.88]
Highest	0.65^**^	[0.17;1.14]
Rho	−0.74	[−0.97;0.20]

CI, confidence interval. ^*^: *p* < 0.05; ^**^: *p* < 0.01; ^***^: *p* < 0.001

^a^ At least one antenatal visit

^b^ Based on the median annual income

^c^ Based on the index of spatial accessibility (ISA)

^d^ FTEs (full-time equivalents) per 100 deliveries

We also computed the correlation between the woman’s level of prenatal education and information about postnatal care and the socioeconomic environment (measured as the median annual income in the municipality). The correlation was significantly positive for both prenatal education and prenatal information regarding postpartum care (coefficient = 0.17, *p* < 0.001, and coefficient = 0.13, *p* < 0.001, respectively). Thus, the higher the median annual income in the municipality, the higher the woman’s level of prenatal education and information about postnatal care. We also estimated the correlation between the accessibility to a midwife and the socioeconomic environment on the subsample of all of the women’s municipalities (*n* = 279). The correlation was significantly positive (coefficient = 0.35, *p* < 0.001). Thus, the higher the median annual income, the greater the accessibility to a midwife. As a sensitivity analysis, we also computed the same correlation at the national level using all French municipalities. The results remained unchanged (Table A3 in the Appendix).

## Discussion

### Key findings

Of the total pool of eligible low-risk women giving birth in hospitals in the Yvelines district, 59.6% of them enrolled and subsequently fully participated in the PRADO program. Controlling for the full set of independent factors, the household’s intrinsic characteristics, the woman’s level of prenatal education and information about postnatal care, and the hospital characteristics and organization impacted enrollment in the PRADO program, while accessibility to health professionals impacted full participation in the PRADO program. Furthermore, the women’s level of prenatal education and information about postnatal care, as well as their accessibility to healthcare professionals correlated with their socioeconomic environment.

### Strengths and limitations

To the best of our knowledge, this study is the first research analysis of access to the PRADO program. On the one hand, our study has many strengths. First, we had access to a large dataset of women eligible for the home-based PRADO program and who had given birth in different hospitals of the Yvelines district. Also, the dataset used for the analysis was checked by the statistics department of the public health insurance of the Yvelines district. Notably, it did not include any missing values for the variables considered in the study. Both the large scale and the high quality of the data allowed us to obtain reliable and accurate results. Secondly, we used many available variables, which enabled us to consider different characteristics of the women throughout their maternity trajectories at the patient, municipality, and hospital levels. This reduced the risk of confusion bias. Thirdly, we considered the entire population of eligible women with respect to the postnatal coordinated care rather than just the subset of women who chose to participate in the PRADO program. This allowed us to address the possible individual self-selection in enrollment in this program, as we employed a Heckman selection model to manage self-selection bias. Fourthly, in order to estimate the accessibility to primary health professionals, we used the index of spatial accessibility (ISA), which made it possible to simultaneously consider healthcare supply, demand, and access. Indeed, this new indicator is more accurate than the usual indices of healthcare accessibility such as access time, access distance, and density by living area.

On the other hand, our analysis could also have some limitations. The study involved women affiliated with a French local public health insurance system. However, the single-payer structure of the French healthcare system (100% of the population) eliminated the potential for program choice to be dictated by the type of health insurance coverage. Moreover, the Yvelines district includes differences in terms of socioeconomic environment and accessibility to health professionals [17]. The diversity of geographic conditions covered by the Yvelines district supports the representativeness of the study and, therefore, the transferability of our results to the French situation. Although we controlled for many of the available covariates, confounding could not be fully ruled out. Indeed, as information regarding the race, ethnicity, and immigration status of the women who enrolled and fully participated in the PRADO program was not available, we could not adjust for it. We used administrative databases for which the law precludes this information from being made available. It would have been necessary to carry out a specific non-administrative survey, but that would have caused a matching problem since the administrative datasets used were de-identified. Therefore, our study could not specifically identify ethnic disparities in the use of the PRADO program. However, some of the women’s characteristics used in the study, such as socioeconomic and location variables, which usually correlated with racial inequities, may capture part of these ethnic disparities, thereby reducing the potential for confounding. We used 2013 datasets as these allowed us to have access to detailed information that was not available with more recent data. Indeed, some of the information used here, such as the number of children in the household, prenatal information regarding postpartum care, and some hospital characteristics, were specifically collected for the year 2013. Furthermore, this 2013 dataset has been specifically checked for study analyses, thereby allowing correction of false and missing information. This quality check substantially improved the reliability of this dataset and thus the validity of our results. Our study was based on statistical analysis of the factors that affect enrollment and full participation in the PRADO program. The use of qualitative or mixed methods studies, particularly when using open-ended interviews, might have captured some possible explanations for our results that this quantitative study could not. Finally, we used the index of spatial accessibility (ISA) that is estimated at the municipality level. However, this multidimensional index is assessed using different parameters of healthcare supply and demand that allows disparities in access to healthcare to be captured at this level, which a standard density indicator will tend to mask [18].

### Interpretation

The first finding of our study was that only a portion of the women eligible for participation in the PRADO program decided to enroll and fully participated in it. During the hospital stay, similar healthcare services are offered to all patients. However, once they have been discharged from the hospital, the patient is expected to be active. They should, therefore, understand the medical recommendations and organize their care pathway accordingly. This raises the role of the patient and their environment in the use of ambulatory healthcare services [19].

We sought then to identify the determinant factors that explain both the initial enrollment and full participation in this postpartum care program. We found that the household’s intrinsic characteristics including demographic and family-context variables, the woman’s level of prenatal education and information about postnatal care, and characteristics and organization of maternity units impacted enrollment in the PRADO program, while we found that accessibility to primary healthcare professionals, particularly accessibility to a midwife, impacted full participation in the PRADO program. Notably, pregnancy in middle age, a low number of children, a high level of prenatal education and information about postnatal care, and some hospital characteristics increased enrollment in the PRADO program, while greater accessibility to a midwife increased full participation in the program. A number of previous studies have reported the same findings for different community medical care services. A US study has observed substantial differences in diagnostic practices across regions that could be explained by patient characteristics but that could also be related to local conditions [20]. A German study has found that differences in demography and the supply of services explained part of the variation in the utilization of ambulatory services for general practitioner, specialist, and psychotherapist consultations [21]. A Swedish study has indicated that demography could explain a considerable part of the regional variation in visits to outpatient specialists, while basic supply-side factors, including the proportion of providers and physicians per inhabitants, could explain part of the regional variation in primary physician visits [22]. Another US study found that patient information regarding integrated care was associated with outpatient utilization but not inpatient utilization [23]. A German study has reported that cancer screening services were significantly higher in areas with higher physician density [24]. Another German study found that supply-side factors accounted for most of the regional inequities in the actual utilization of outpatient health services [25].

Finally, we observed that the woman’s level of prenatal education and information about postnatal care and the area-based accessibility to professionals correlated with the socioeconomic environment. That is to say, a higher level of prenatal education and information about postnatal care and a higher level of accessibility to a midwife correlated with a higher socioeconomic environment. Past analyses have shown that socioeconomic factors impact women’s participation in prenatal education during their maternity pathway [26]. Furthermore, a number of recent studies have reported a relationship between local socioeconomic conditions, the distribution of healthcare professionals, and the efficiency of healthcare provision [27, 28]. The French healthcare system, although it has a high level of favorable health outcomes and patient satisfaction, still suffers from socioeconomic, geographic, and cultural health inequalities, including inequity in terms of access to relevant health information and to healthcare professionals, which could both be major barriers to health equity [29]. This is also the current situation in many other developed countries [30, 31]. Moreover, the issue of socioeconomic disparities is the focus of much attention in reproductive health and still needs to be addressed more thoroughly [26, 32].

### Policy implication and prospects

In recent years, ambulatory care has been promoted as an effective healthcare service that could replace unnecessary hospital stay care, which should only be reserved for patients requiring continuous medical surveillance. This ambulatory shift has accentuated the need for adequate investment in patient empowerment and community infrastructure to ensure continuity of care.

The results of our study may have a number of public policy implications. A straightforward implication would be that health policies promoting the level of information of patients or accessibility to primary health professionals, which are possibly editable components of the healthcare system, could lead to better follow-up of community care, especially after hospital discharge. Furthermore, since these two factors differ across different socioeconomic environments, patient and spatial needs along with social characteristics should be considered in order to improve the allocation of medical services.

The French public insurance system recently implemented financial incentives to promote the appointment and retention of independent health professionals, including midwives, in areas where the supply of care is insufficient or the access to care is difficult [33]. Future research should study the impact of such experimentations on the use of outpatient care. Moreover, other interventions that may enhance access to healthcare professionals should be investigated. For instance, in the National Health Service, one study on home-based postnatal care that involved a series of home visits reported that small increases in travel time of midwives boosted continuity of care and, therefore, access to community midwifery services [34]. Financial incentives can also be used to promote the degree to which women are informed. As an example, the financial aid usually allocated to future parents to prepare for the arrival of their child, which is more important for the socially disadvantaged population, could be modulated according to the degree of the woman’s use of prenatal care.

## Conclusion

In conclusion, enrollment in the PRADO program was determined primarily by the household intrinsic characteristics, the woman’s level of prenatal education and information about postnatal care, and hospital characteristics and organization; while full participation (adherence) in the program was determined primarily by the accessibility to health professionals. These findings emphasize the importance of improving the level of information and the accessibility to professionals, which are both lower for less affluent women, as potential ways to get more women to use community postnatal care. Future high-quality studies are needed to evaluate the effect of interventions that increase patient empowerment or healthcare access on postnatal care use.

## Supplementary Information

**Additional file 1 Table S1.** Factors associated with full participation in the home-based postnatal coordinated care (PRADO) for the women enrolled in this program. Probit regression model. Hospital characteristics included (*n* = 2859).

**Additional file 2 Table S2.** Factors associated with full participation in the home-based postnatal coordinated care (PRADO) for all of the women eligible for this program. Probit Heckman regression model allowing self-selection into women enrolled in this program. Results of the selection equation (*n* = 4189).

**Additional file 3 Table S3.** Correlation between accessibility to health professionals and the socioeconomic environment. All French municipalities (*n* = 32,948).

## Data Availability

The data that support the findings of this study are available from the public health insurance agency of the Yvelines district but restrictions apply to the availability of these data, which were used under license for the current study, and so are not publicly available. Data are however available from the authors upon reasonable request and with permission of the public health insurance agency of the Yvelines district.
