# Supplementary material for: Access-to-care: evidence from home-based postnatal coordinated care after hospital discharge
Source: BMC Health Serv Res. 2021 Oct 22;21:1137. doi: 10.1186/s12913-021-07151-3 (PMC8532282; doi:10.1186/s12913-021-07151-3)
Supplement: Supplementary file 1 — Additional file 1 Table S1. Factors associated with full participation in the home-based postnatal coordinated care (PRADO) for the women enrolled in this program. Probit regression model. Hospital characteristics included (n = 2859). [file 12913_2021_7151_MOESM1_ESM.docx]

**Table A1.** Factors associated with full participation in the home-based postnatal coordinated care (PRADO) for the women enrolled in this program. Probit regression model. Hospital characteristics included (n = 2,859).

**Coefficient** [**95% CI**]

**Household characteristics**

Woman's age at pregnancy (years)

18 - 23 -0.02 [-0.29;0.26]

24 - 29 0.09 [-0.04;0.21]

30 - 35 reference

36 - 41 0.06 [-0.08;0.20]

≥ 42 -0.02 [-0.47;0.42]

Number of children

1 reference

2 -0.12 [-0.28;0.04]

3 -0.13 [-0.34;0.07]

≥ 4 -0.22 [-0.50;0.05]

Woman's healthcare coverage

Policyholder reference

Beneficiary -0.16 [-0.37;0.05]

**Prenatal care**

Antenatal visits

0 - 5 -0.02 [-0.23;0.20]

6 - 7 reference

≥ 8 0.12 [-0.04;0.27]

Follow-up by a gynecologist^a^

No reference

Yes 0.02 [-0.12;0.15]

Follow-up by a general practitioner^a^

No reference

Yes -0.12 [-0.31;0.08]

Follow-up by a midwife^a^

No reference

Yes 0.00 [-0.16;0.15]

Hospital follow-up^a^

No reference

Yes 0.06 [-0.12;0.23]

Community follow-up^a^

No reference

Yes 0.14 [-0.08;0.36]

Obstetric ultrasound

0 - 1 -0.03 [-0.23;0.16]

2 - 3 reference

≥ 4 -0.15^*^ [-0.29;-0.01]

Prenatal education

No reference

Yes 0.21^**^ [0.06;0.36]

Prenatal information regarding postpartum

No reference

Yes 0.11 [-0.09;0.30]

**Postnatal care**

Hospital readmission

No reference

Yes -0.72^***^ [-1.09;-0.35]

**Municipality characteristics**

Location

Urban reference

Rural 0.00 [-0.26;0.26]

Household deprivation^b^

Least deprived reference

Less deprived 0.11 [-0.16;0.38]

More deprived 0.15 [-0.18;0.48]

Most deprived -0.14 [-0.47;0.20]

Accessibility to a gynecologist^c^

Lowest reference

Low -0.21 [-0.46;0.03]

High -0.19 [-0.59;0.21]

Highest 0.15 [-0.40;0.70]

Accessibility to a general practitioner^c^

Lowest reference

Low 0.23 [-0.17;0.63]

High 0.19 [-0.24;0.61]

Highest 0.39 [-0.10;0.88]

Accessibility to a midwife^c^

Lowest reference

Low 0.64^***^ [0.33;0.96]

High 0.68^***^ [0.32;1.05]

Highest 0.72^**^ [0.24;1.21]

**Hospital characteristics**

Funding

Public reference

Private 0.05 [-0.40;0.51]

University status

Non-teaching reference

Teaching -0.03 [-0.28;0.23]

Level of care

No neonatology unit reference

Neonatology unit -0.23 [-0.51;0.05]

Neonatal intensive care unit 0.15 [-0.24;0.54]

Obstetricians^d^  0.84 [-0.83;2.51]

Midwives^d^ 0.27 [-0.59;1.13]

Day of delivery

Working reference

Non-working -0.16 [-0.47;0.16]

Day of discharge

Working reference

Non-working -0.31 [-0.64;0.01]

CI, confidence interval. ^*^: p < 0.05; ^**^: p < 0.01; ^***^: p < 0.001

^a^ At least one antenatal visit

^b^ Based on the median annual income

^c^ Based on the index of spatial accessibility (ISA)

^d^ FTEs (full-time equivalents) per 100 deliveries
