# Supplementary material for: Access-to-care: evidence from home-based postnatal coordinated care after hospital discharge
Source: BMC Health Serv Res. 2021 Oct 22;21:1137. doi: 10.1186/s12913-021-07151-3 (PMC8532282; doi:10.1186/s12913-021-07151-3)
Supplement: Supplementary file 2 — Additional file 2 Table S2. Factors associated with full participation in the home-based postnatal coordinated care (PRADO) for all of the women eligible for this program. Probit Heckman regression model allowing self-selection into women enrolled in this program. Results of the selection equation (n = 4189). [file 12913_2021_7151_MOESM2_ESM.docx]

**Table A2.** Factors associated with full participation in the home-based postnatal coordinated care (PRADO) for all of the women eligible for this program. Probit Heckman regression model allowing self-selection into women enrolled in this program. Results of the selection equation (n = 4,189).

**Coefficient** [**95% CI**]

**Household characteristics**

Woman's age at pregnancy (years)

18 - 23 -0.32^***^ [-0.45;-0.18]

24 - 29 -0.05 [-0.14;0.04]

30 - 35 reference

36 - 41 0.07 [-0.03;0.18]

≥ 42 -0.41^**^ [-0.69;-0.14]

Number of children

1 reference

2 -0.10 [-0.20;0.01]

3 -0.29^***^ [-0.41;-0.18]

≥ 4 -0.31^***^ [-0.47;-0.15]

Woman's healthcare coverage

Policyholder reference

Beneficiary -0.15^*^ [-0.28;-0.01]

**Prenatal care**

Antenatal visits

0 - 5 -0.05 [-0.19;0.09]

6 - 7 reference

≥ 8 -0.02 [-0.13;0.09]

Follow-up by a gynecologist^a^

No reference

Yes -0.08 [-0.23;0.06]

Follow-up by a general practitioner^a^

No reference

Yes 0.09 [-0.03;0.22]

Follow-up by a midwife^a^

No reference

Yes -0.09 [-0.23;0.05]

Hospital follow-up^a^

No reference

Yes -0.07 [-0.23;0.10]

Community follow-up^a^

No reference

Yes -0.06 [-0.23;0.10]

Obstetric ultrasound

0 - 1 -0.07 [-0.22;0.08]

2 - 3 reference

≥ 4 0.03 [-0.09;0.14]

Prenatal education

No reference

Yes 0.46^***^ [0.36;0.56]

Prenatal information regarding postpartum

No reference

Yes 0.71^***^ [0.50;0.92]

**Municipality characteristics**

Location

Urban reference

Rural 0.08 [-0.13;0.29]

Household deprivation^b^

Least deprived reference

Less deprived 0.23 [-0.10;0.57]

More deprived 0.07 [-0.28;0.42]

Most deprived -0.05 [-0.41;0.31]

Accessibility to a gynecologist^c^

Lowest reference

Low -0.07 [-0.23;0.09]

High 0.12 [-0.12;0.35]

Highest 0.56^**^ [0.23;0.90]

Accessibility to a general practitioner^c^

Lowest reference

Low 0.01 [-0.29;0.31]

High 0.07 [-0.24;0.38]

Highest 0.06 [-0.26;0.37]

Accessibility to a midwife^c^

Lowest reference

Low 0.16 [-0.07;0.40]

High 0.16 [-0.10;0.41]

Highest -0.10 [-0.41;0.21]

**Hospital characteristics**

Funding

Public reference

Private -0.40^*^ [-0.73;-0.08]

University status

Non-teaching reference

Teaching 0.41^***^ [0.23;0.59]

Level of care

No neonatology unit reference

Neonatology unit -0.46^***^ [-0.69;-0.23]

Neonatal intensive care unit -0.49^**^ [-0.84;-0.13]

Obstetricians^d^ -2.02^**^ [-3.47;-0.58]

Midwives^d^ -0.81^**^ [-1.41;-0.21]

Day of delivery

Working reference

Non-working 0.23 [-0.05;0.51]

Day of discharge

Working reference

Non-working -0.28^*^ [-0.51;-0.06]

CI, confidence interval. ^*^: p < 0.05; ^**^: p < 0.01; ^***^: p < 0.001

^a^ At least one antenatal visit

^b^ Based on the median annual income

^c^ Based on the index of spatial accessibility (ISA)

^d^ FTEs (full-time equivalents) per 100 deliveries
