# Supplementary material for: Access-to-care: evidence from home-based postnatal coordinated care after hospital discharge
Source: BMC Health Serv Res. 2021 Oct 22;21:1137. doi: 10.1186/s12913-021-07151-3 (PMC8532282; doi:10.1186/s12913-021-07151-3)
Supplement: Supplementary file 3 — Additional file 3 Table S3. Correlation between accessibility to health professionals and the socioeconomic environment. All French municipalities (n = 32,948). [file 12913_2021_7151_MOESM3_ESM.docx]

**Table A3.** Correlation between accessibility to health professionals and the socioeconomic environment. All French municipalities (n = 32,948).

**Accessibility to Accessibility to Accessibility to a gynecologist^a^ a general practitioner^a^ a midwife^a^**

**Socioeconomic environment**

Household median annual income 0.39^***^ 0.02^***^ 0.12^***^

^*^: p < 0.05; ^**^: p < 0.01; ^***^: p < 0.001

^a^ Based on the index of spatial accessibility (ISA)
